# Microbiota in Mild Inflammatory Bowel Disease (IBD) Can Be Modulated by Beta-Glucans and Mannanoligosaccharides: A Randomized, Double-Blinded Study in Dogs

**DOI:** 10.3390/vetsci11080349

**Published:** 2024-08-01

**Authors:** Andressa Rodrigues Amaral, Mariana Fragoso Rentas, Thais Caroline Taveira Rosa, Thais Araújo Esteves Pereira, Pedro Henrique Marchi, Fabio Alves Teixeira, Fernando de Oliveira Roberti Filho, Thaila Cristina Putarov, Bruno Cogliati, Thiago Henrique Annibale Vendramini, Júlio Cesar de Carvalho Balieiro, Marcio Antonio Brunetto

**Affiliations:** 1 Veterinary Nutrology Service, Veterinary Teaching Hospital, School of Veterinary Medicine and Animal Science, University of Sao Paulo, Sao Paulo 05508-270, Brazil; andressa.rodrigues.amaral@usp.br (A.R.A.); thaist.rosa97@gmail.com (T.C.T.R.); shukudai.vet@gmail.com (T.A.E.P.); fabioa14@hotmail.com (F.A.T.); 2 Pet Nutrology Research Center, School of Veterinary Medicine and Animal Science, University of Sao Paulo, Pirassununga 13635-900, Brazil; mvmarirentas@gmail.com (M.F.R.); pedro.henrique.marchi@usp.br (P.H.M.); balieiro@usp.br (J.C.d.C.B.); 3 Independent Researcher, 35238 Rennes, France; 4 Biorigin, Lençóis Paulista 18680-900, Brazil; fthaila.putarov@biorigin.net; 5 Pathology Department, School of Veterinary Medicine and Animal Science, University of Sao Paulo, Sao Paulo 05508-270, Brazil; bcogliati@usp.br

**Keywords:** chronic enteropathy, dog, dysbiosis, nutrition, prebiotic

## Abstract

**Simple Summary:**

Chronic inflammation in the intestine that can lead to detrimental changes in the composition of bacteria living in the gut, which includes increasing disease-causing bacteria and decreasing those that control them and are beneficial to the host. This shift may exacerbate and complicate treatment efforts. Intestinal bacteria are important to control inflammation and, when the cause of inflammation cannot be completely withdrawn, medications and dietary adjustments become necessary to manage symptoms. Prebiotics are fiber sources that nourish beneficial bacteria and can be provided in food or concentrated in capsules. This study found that the daily intake of beta-glucans (a type of prebiotic) for 60 days positively modulated the gut microbiota in dogs with this condition. The patients receiving prebiotics did not experience any disease relapse over a period of 6 months.

**Abstract:**

Inflammatory bowel disease (IBD) in dogs is the most common chronic gastrointestinal disease in dogs. Its etiology evolves an aberrant immunological response towards food antigens and indigenous bacteria in the gut bacteria and, consequently, dysbiosis. Prebiotics provide substrates for the growth of beneficial bacteria and promote the production of beneficial fermentation products. This study aimed to evaluate the effects of oral supplementations of beta-glucans and mannanoligosaccharides (MOSs) over 60 days in fecal microbiota and fecal concentrations of fermentation products in dogs with mild IBD. Eighteen dogs with mild IBD were divided into three experimental groups in a blinded and randomized manner: A—dogs received 0.1% of a beta-glucan-based prebiotic, B—dogs received 0.1% of a MOS + beta-glucan-based prebiotic, and C—dogs received 0.1% of a placebo. Fecal microbiota was analyzed using the latest generation 16S rRNA sequencing (Illumina^®^). Relative abundances of each taxon were analyzed using a generalized linear model, and fermentation products using a mixed model. A significance level of p was used. The prebiotics positively modulated the bacterial population of Firmicutes and Bacteroidetes. Treatment A improved alpha diversity and populations of beneficial bacteria. Beta-glucan supplementation for 60 days had beneficial effects on modulating intestinal microbiota in dogs with mild IBD.

## 1. Introduction

Inflammatory bowel disease (IBD) is the most common cause of chronic gastrointestinal (GI) disease in dogs, with a multifactorial etiology involving genetic changes that impair the immune response and normal recognition of bacterial and food antigens [1]. Treatment evolves pharmacological and nutritional approaches such as the use of immunosuppressive therapy and a hypoallergenic diet to minimize the clinical signs [2,3].

Recent studies with dogs have already reported a close relationship between intestinal dysbiosis, its fermentation products, and IBD, which can either promote remission or induce mucosal inflammation [4,5,6,7]. The gastrointestinal tracts of humans, cats, and dogs harbor trillions of microorganisms, including bacteria, archaea, fungi, and protozoa. These microbiota plays crucial roles in host health by supporting the immune system, producing metabolites with nutritional and signaling functions, maintaining intestinal barrier integrity, and resisting pathogenic colonization. Dysbiosis, observed in conditions like acute and chronic gastrointestinal inflammatory diseases or following antibiotic use, impacts host health and metabolism [8].

The term dysbiosis refers to changes in microbiota diversity and functional changes, such as alterations in the production of bacterial metabolites. This scenario is commonly observed in many chronic diseases and can be assessed through many tools [9].

Short-chain fatty acids (SCFAs), including acetate, propionate, and butyrate, are major end products of dietary fiber fermentation by gut bacteria. Their role in health and disease was previously evaluated, considering their relationship with digestive microbiota and their effects on the immune system and gastrointestinal motility. Reduced fecal SCFA levels have been observed in various chronic digestive conditions, including IBD, and among populations at high risk for colon cancer [10,11,12].

In response to dysbiosis and altered fermentation products, emerging therapies such as fecal microbiota transplantation (FMT) have been developed. FMT involves transferring fecal material from healthy donors to recipients to modulate their intestinal microbiome, administered through methods like enema, colonoscopy, or oral capsules [13]. Its application in veterinary medicine, particularly in dogs, is relatively recent.

In the light of the need to restore the altered microbiota, fecal transplantation has been shown to promote the growth of butyrate-producing bacteria, which stimulate regulatory T cells, enhance interleukin-10 production, and modulate mucosal immune responses to reduce inflammation, for example. Additionally, bacteriophages present in donor feces may contribute to these beneficial effects [13].

In veterinary medicine, initial studies suggest promising applications of FMT in acute gastrointestinal disorders in dogs, such as parvovirus infections, where it has demonstrated potential in reducing symptom duration and hospital stays [13]. 

While fecal transplantations are still being researched, the promising effects of prebiotics in microbiota support their use as a complementary alternative for treatment in diseases influenced by dysbiosis, such as IBD [14,15,16], and consists of the application of a non-invasive approach to modulate fecal microbiota that can still be introduced in daily food. 

Beta-glucans and mannanoligosaccharides (MOSs) are prebiotics derived from yeasts with the potential to control intestinal inflammation in humans, dogs, and rodent models of IBD [17,18]. However, limitations related to the lack of individualized treatments, varied diets, and heterogenous experimental groups compromise the interpretation of their real effectiveness, so research that can first identify what they can or cannot do in controlled conditions is still being conducted.

In a systematic review published by this research group [19], only two studies have evaluated the effects of beta-glucan on inflammatory bowel disease. Translating this application for clinical use remains a challenge that needs to be addressed by understanding the effects of this prebiotic, its appropriate dosage, and its optimal timing without the confounding influence of medications or relapsing episodes.

Therefore, this study aimed to assess the effects of orally supplemented beta-glucan and MOSs on fecal microbiota and the concentration of short-chain fatty acids (SCFAs) in dogs with mild IBD using a randomized and double-blinded study design.

## 2. Materials and Methods

### 2.1. Experimental Design

This prospective, randomized, double-blinded clinical trial was approved by the Animal Use Ethics Committee of the School of Veterinary Medicine and Animal Science (FMVZ) of the University of São Paulo (CEUA 1102270218) and by the Council of the FMVZ Veterinary Hospital (HOVET).

Eighteen dogs with IBD, diagnosed according to the 2010 ACVIM Consensus Statement [20], were selected from routine cases at HOVET. Dogs were required to be in a mild phase of the disease based on the canine chronic enteropathy clinical activity index (CCECAI) scale (grades between 0 and 3) [21], and without concomitant illnesses. Screening tests for endoparasites, hypoadrenocorticism, pancreatitis, and exocrine pancreatic insufficiency (EPI) were performed during the diagnostic phase. Exclusion criteria included recent use (within 30 days) of prebiotics, probiotics, corticosteroids, antibiotics and any need to modify or include treatments during the study period. The dogs underwent a 60-day trial to identify food allergies before being selected for the study. As part of this process, antibiotic therapy had been discontinued for at least 90 days prior to selection, though some patients may have received this treatment before that period.

All dogs received the same hypoallergenic diet throughout the study and in the 30-day acclimation phase preceding the study. The dogs were randomized into three experimental groups: A—dogs received 0.1% of a 1.3/1.6 beta-glucan-based prebiotic (concentration = 60%), B—dogs received 0.1% of a MOS + 1.3/1.6 beta-glucan-based prebiotic (concentration = 20% and 25%, respectively), and C—dogs received 0.1% of a grounded form of the hypoallergenic diet (placebo). Treatments were encapsulated (enteric capsules) and administered orally once daily for 60 days.

At the beginning (T0) and end (T60) of the experimental period, blood and fecal samples were collected after a 12 h fast for laboratory analysis. Whole blood was analyzed for hematological parameters and serum was analyzed for urea, creatinine, total protein count, albumin, alanine aminotransferase, alkaline phosphatase, cholesterol, and triglycerides at the HOVET laboratory. Cobalamin, folate, canine pancreas-specific lipase (Spec cPL), canine trypsin-like immunoreactivity (cTLI), and C-reactive protein (CRP) were analyzed at Idexx^®^ laboratory, São Paulo, Brazil.

Fresh fecal samples were collected by spontaneous defecation using sterile gloves. A portion was stored at −80 °C for microbiota analysis and another was used for the analysis of short-chain and branched-chain fatty acids (BCFAs). Fecal score (FS) was classified using a visual scale at the time of collection.

The dogs were re-evaluated every 20 days (e.g., T0, T20, T40, and T60), weighed, and owners were interviewed for general follow-up. The CCECAI score was applied and FSs were recorded with the help of an illustrative scheme from Carciofi et al. (2008) scale [22]. A physical examination was conducted, including the evaluation of the body condition score (BCS) according to Laflamme (1997) [23] and the muscle mass score (MMS) according to Michel et al. (2011) [24]. 

Food intake was calculated to provide the recommended daily calorie intake for inactive adult dogs according to the NRC (2006) [25]. Dogs were monitored to maintain a stable body weight during the study period, with adjustments made to calorie intake if a weight loss or weight gain of at least 5% was observed.

### 2.2. Microbiota Analysis

Microbiota analyses were carried out on BPI technology (Botucatu, São Paulo, Brazil) using the Illumina MiniSeq next-generation sequencing system (Illumina^®^, San Diego, CA, USA). Sequences were filtered based on quality and clustered into operational taxonomic units (OTUs) at 97% similarity. A representative sequence from each OTU was used to build a phylogenetic tree, which was used for beta diversity analysis. The relative abundance of each OTU in the samples was used for alpha and beta diversity analysis.

### 2.3. Short and Branched-Chain Fatty Acid Concentrations

Fresh fecal samples were homogenized and weighed. For lactic acid quantification, three grams of feces were diluted in 0.9 mL of distilled water. For short-chain and branched-chain fatty acids, three grams of feces were diluted in 0.9 mL of 16% formic acid. The mixtures were refrigerated for seven days, with daily homogenization, and were subsequently centrifuged three times for 15 min at 15 °C at 5000 rpm. Lactic acid levels were quantified using the spectrophotometry method at 565 nm (range 500 to 570 nm) following the methodology described by Pryce (1969) [26]. SCFAs and BCFAs were quantified using gas chromatography, according to Erwin et al., 1961 [27]. These analyses were conducted at the Multi-User Laboratory of Animal Nutrition and Bromatology of the Department of Animal Nutrition and Production, FMVZ/USP.

### 2.4. Prebiotic Composition

The chemical contents of the prebiotics are described in Table 1.

### 2.5. Statistics

Results were analyzed using the Statistical Analysis System, version 9.4 (SAS Institute Inc., Cary, NC, USA). Variables including hematological and serum quantifications, body weight, BCS, MMS, FS, and CCECAI were assessed using a mixed model. This model incorporated fixed effects for treatment and treatment-by-time interaction. All analyzes were performed using the PROC MIXED procedure of SAS.

For microbiota evaluation, the abundances observed for each phylum, class, family, and genus were analyzed using a mixed, generalized linear model with a binomial distribution. The statistical model included the fixed effects of treatments, treatment-by-time interaction, and a repeated measures structure over time.

Analysis of SCFAs, BCFAs, and LA involved the comparison of average results using a generalized mixed model. This model included the fixed effect for treatments, treatment-by-time interaction, and repeated measures over time.

The proportion of each OTU (operational taxon unit) in each sample was used for alpha and beta diversity analyses. Average percentages of taxon abundance and mean absolute values of alpha and beta diversity were compared by the Tukey test and values of *p* ≤ 0.05 were considered significant.

## 3. Results

The study included 18 dogs with IBD, comprising 8 males and 10 females, with a mean age of 7.2 ± 3.64 years, an average body weight (BW) of 13.88 ± 9.86 kg, a mean BCS of 5.28 ± 0.75, and a mean MMS of 2.83 ± 0.38.

**Figure 1:**
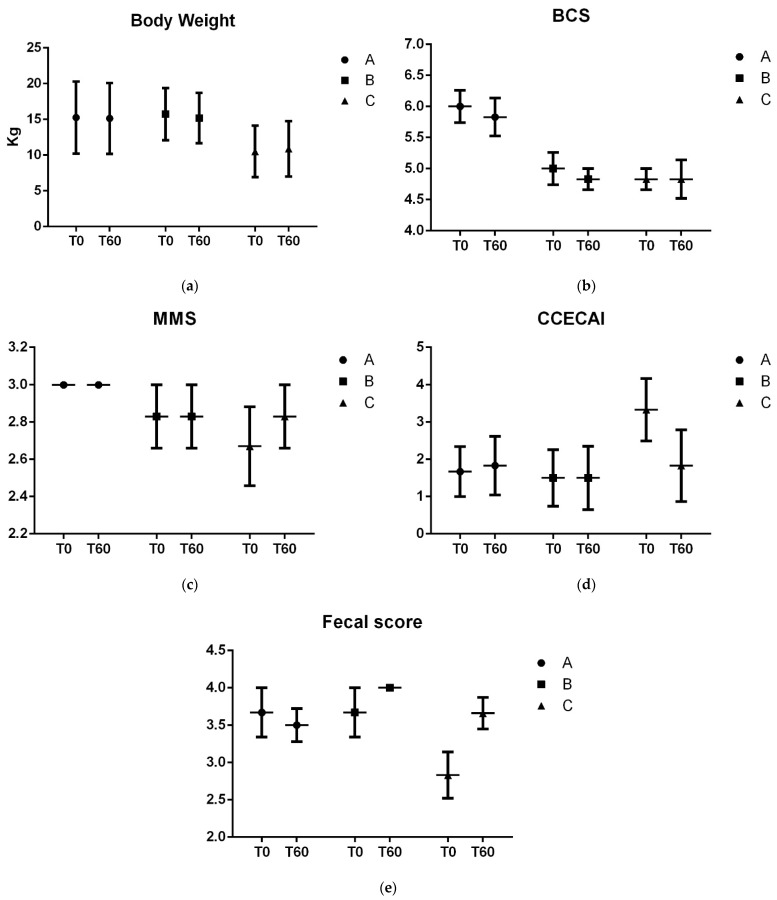
Mean values of BW (**a**), BCS (**b**), MMS (**c**), CCECAI (**d**), FS (**e**) at T0 and T60. A: 1.3/1.6 beta-glucan; B: MOS + 1.3/1.6 beta-glucan; C: placebo.

None of the treatments changed for the BW, BCS, MMS, FS, and CCECAI over time, indicating stable clinical statuses during the experiment, as expected (Figure 1).

Results of the blood variables analysis are presented in Table 2. In general, none of the patients exhibited alterations in blood check-up variables that could compromise their participation in the study. Animals from group A had, on average, serum alkaline phosphatase, triglyceride, and cholesterol concentrations above the reference range. Differences between groups were observed for cholesterol levels. Additionally, both groups A and C had serum CRP concentrations higher than the reference range, without differing from group B. Platelet counts also were different between treatment groups, with higher mean values in group A, lower values in group B, and intermediate values in group C, which it did not differ from the other groups. 

Dogs in group C exhibited higher mean monocyte count values at T60 (570.00 ± 232.70/μL) compared to T0 (366 ± 149.72/μL) (*p* = 0.0331).

Platelet-to-lymphocyte (PLR) and monocyte-to-lymphocyte (MLR) ratios were calculated for A, B, and C and results were that the PLR of group A was 278, of group B was 173, of C was 188, and that the MLR of group A was 0.37, of group B was 0.2, and of C was 0.2.

### 3.1. Fecal Lactic Acid, Short and Branched-Chain Fatty Acids

**Figure 2:**
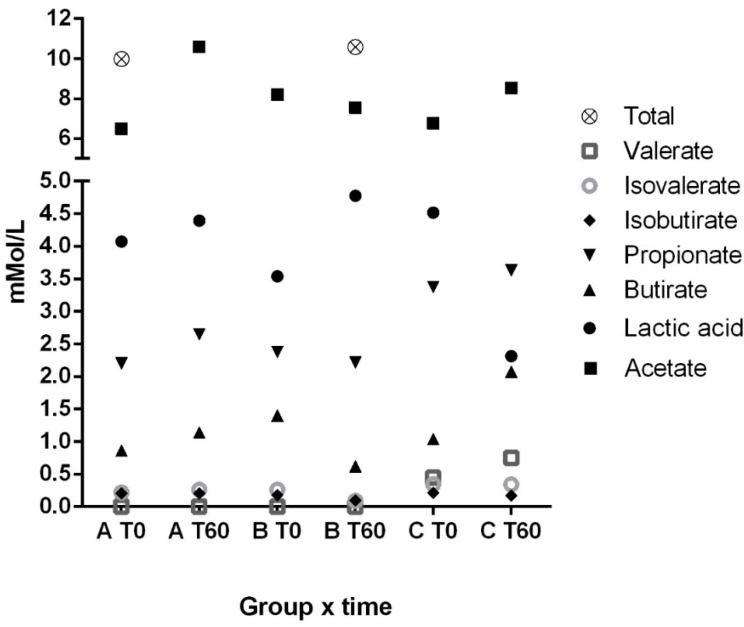
Fecal concentration of short and branched-chain fatty acids in the experimental groups. A: 1.3/1.6 beta-glucan; B: 1.3/1.6 beta-glucan + MOS; C: placebo.

Results for lactic acid, SCFAs, and BCFAs are illustrated in Figure 2. No differences were found for these variables between treatments and in treatment-by-time interactions.

### 3.2. Gut Microbiota

When characterizing the gut microbiota, rarefaction curves indicated the sequencing effort with a tendency for stabilization and indicated that the sampling effort was sufficient for evaluating the bacterial community. Analysis of the Shannon alpha diversity index (Table 3) revealed an interaction between time and treatment (*p* = 0.05) where the average values for group A increased at T60 compared to T0. No differences were found for OTU counts.

**Figure 3:**
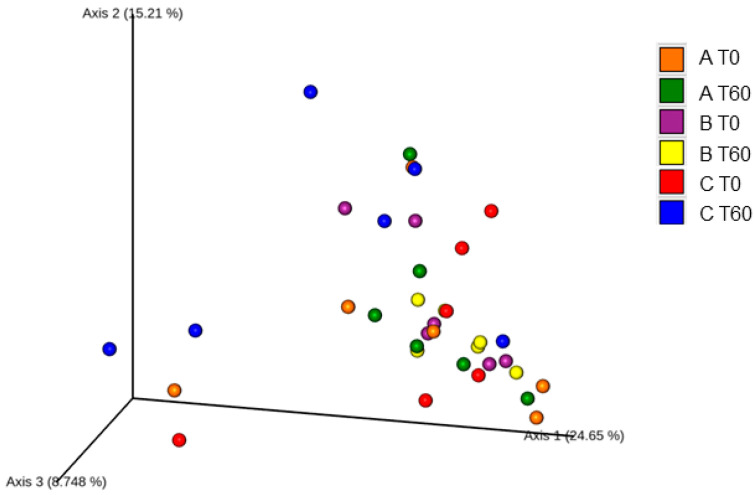
Three-dimensional analysis of the main components with the absolute data obtained by sequencing the samples of the three experimental groups at T0 and T60. A: 1.3/1.6 beta-glucan; B: 1.3/1.6 beta-glucan + MOS; C: placebo.

Beta diversity was assessed using principal component analysis (PCA) (Figure 3) and showed a tendency to group experimental treatments on the same axis. However, b-paired comparisons in the PERMANOVA analysis revealed differences in the microbiome between group B at T0 (*p* = 0.032) and T60 (*p* = 0.014) compared to group C at T60.

Bacterial taxa were compared when the referring taxa were represented in all groups at both T0 and T60, resulting in 5 phyla, 8 classes, 13 families, and 15 genera with valid values for statistical analysis. 

Regarding phylum, class, family, and genera, respectively, Firmicutes had the highest average relative abundance (84%) and Bacteroidetes had the lowest (0.2%); clostridia had the highest average relative abundance (38%) and Actinobacteria, the lowest (0.08%); the Lachnospiraceae family had the highest average relative abundance (24%) and Pseudomonadaceae had the lowest (0.01%); and Streptococcus had the highest average relative abundance (18%) and the genus Pseudomonas had the lowest (0.01%).

**Figure 4:**
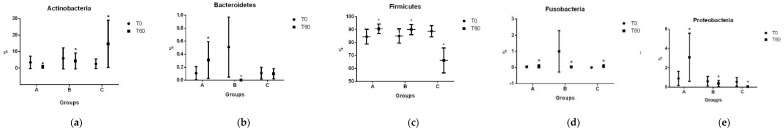
Average percentages of phyla of *Actinobacteria* (**a**), *Bacteroidetes* (**b**), *Firmicutes* (**c**), *Fusobacteria* (**d**), and *Proteobacteria* (**e**) at T0 and T60 in experimental groups. A: 1.3/1.6 beta-glucan; B: 1.3/1.6 beta-glucan + MOS; C: placebo; * *p* < 0.05.

**Figure 5:**
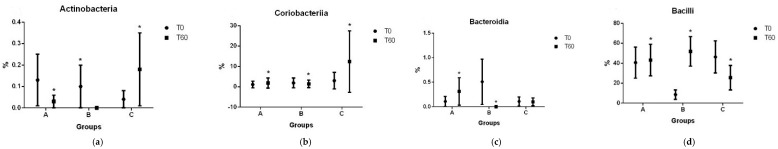

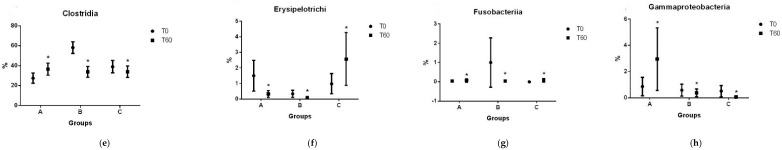
Average percentages of classes of Actinobacteria (**a**), Coriobacteria (**b**), Bacteroidia (**c**), Bacilli (**d**), Clostridia (**e**), Erysipelotrichi (**f**), Fusobacteria (**g**), and Gammaproteobacteria (**h**). A: 1.3/1.6 beta-glucan; B: 1.3/1.6 beta-glucan + MOS; C: placebo; * *p* < 0.05.

**Figure 6:**
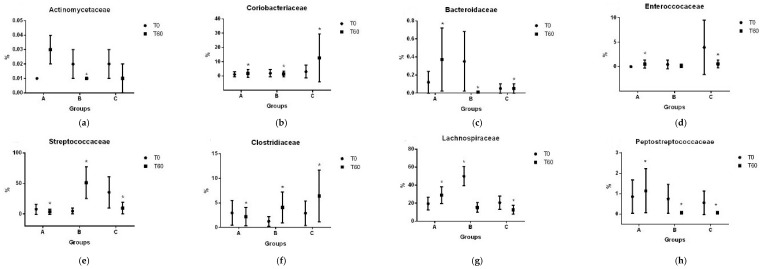

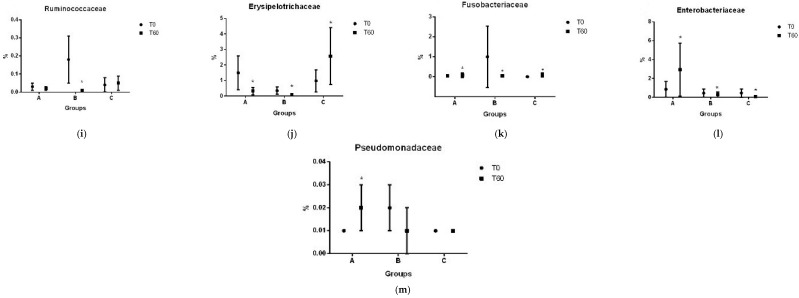
Average percentages of families of Actinomycetaceae (**a**), Coriobacteriaceae (**b**), Bacteroidaceae (**c**), Enterococcaceae (**d**), Streptococcaceae (**e**), Clostridiaceae (**f**), Lachnospiraceae (**g**), Peptostreptococcaceae (**h**), Ruminococcaceae (**i**), Erysipelotrichaceae (**j**), Fusobacteriaceae (**k**), Enterobacteriaceae (**l**), and Pseudomonadaceae (**m**). A: 1.3/1.6 beta-glucan; B: 1.3/1.6 beta-glucan + MOS; C: placebo. * *p* < 0.05.

**Figure 7:**
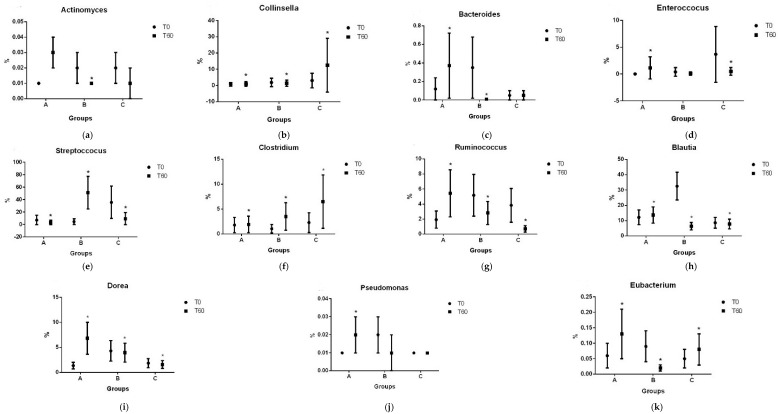
Average percentages of genera of *Actinomyces* (**a**), *Collinsella* (**b**), *Bacteroides* (**c**), *Enterococcus* (**d**), *Streptococcus* (**e**), *Clostridium* (**f**), *Ruminococcus* (**g**), *Blautia* (**h**), *Dorea* (**i**), *Pseudomonas* (**j**), and *Eubacterium* (**k**). A: 1.3/1.6 beta-glucan; B: 1.3/1.6 beta-glucan + MOS; C: placebo. * *p* < 0.05.

Results for each bacteria taxa are illustrated in Figure 4, Figure 5, Figure 6 and Figure 7.

Regarding the differences found in treatment by time interactions of the core bacteria, treatments A and B resulted in the growth of the phylum *Firmicutes* (+7 and +6%, respectively), although only treatment A resulted in an increase in the population of *Bacteroidetes* (+182%) and treatment B had a 100% reduction.

Treatment A promoted the growth of *Proteobacteria* populations (+243%) whereas groups B and C had a reduction (−38% and −89%).

Finally, the class clostridia increased in group A (+33%) but decreased in groups B and C (−41.8% and 12.7%). The class Erysipelotrichia decreased in A and B (−78% and −73%) and increased in C (+162%).

## 4. Discussion

According to the CCECAI, patients graded 0–5 based on clinical signs, albumin concentrations, and pruritus are classified as having a mild disease and do not require medication because the symptoms are mild or absent (even in light of the history and biopsy confirming the chronic enteropathy). Therefore, it is preferable to assess the individual effect of the prebiotic without the interference of medication. In our study, if a patient experiences a relapse at any point, which is anticipated as this disease is incurable, they are removed from the study to receive appropriate treatment and because they would no longer fall within the “controlled” range (0–5). 

In this study, all dogs maintained their previous mild state of IBD with no relapsing of acute GI signs. No changes were observed in physical variables, fecal score, and chronic enteropathy indices, which constituted the primary goal of this study. 

Results of blood check-up analysis demonstrated a difference in platelet count among the experimental groups. A study by Mehain et al. (2019) [28] indicated an association between this variable and the CCECAI, proposing using platelet indices as a potential indirect biomarker of IBD in dogs. Although, in light of the homogeneity of our groups, such a correlation was not identified. Further analysis of the specific platelet component’s distribution (which is the specific platelet component that correlated with CCECAI in the Mehain’s study) is needed.

Ridgway et al. (2001) [29] reported that 2.5% of dogs with IBD in their study had subclinical thrombocytopenia, which normalized with treatment and did not correlate with the severity of intestinal inflammation. Despite these differences, the platelet count was within the normal range for the species, and thrombocytopenia was not observed. 

Similar findings were observed for serum cholesterol, where differences were observed differences between groups, but without clinical significance. A single patient in group A had hypercholesterolemia and severe hypertriglyceridemia (>270 mg/dL and >100 g/dL, respectively), and its inclusion raised the average for this group. This patient was properly treated with a hypolipidemic medication to remain in the study. Dyslipidemia is a common finding in human patients with IBD and it can be attributed to the elevation of inflammatory cytokines such as TNF-α, interferon C, and interleukin-1 that inhibit lipoprotein lipase activity [30,31].

Serum concentrations of CRP and AP also exceeded the reference range in some dogs included in the study. Although, these are also changes justified by the nature of the inflammatory process of the high storage of AP in intestinal cells, potentially increasing in GI injuries [32,33]. The absence of differences in these variables between groups corroborates the homogenous inflammatory state among them. 

Studies with humans have already observed monocytosis in patients with ulcerative colitis and Chron’s disease since, in these cases, this cell type is evolved in acute inflammations [34,35]. In a recent retrospective study by Marchesi et al. [36], it was found that monocyte count (odds ratio = 1.29), the MLR (odds ratio = 8.07), and the PLR (odds ratio = 4.35) were good predictors of IBD in a cohort of 60 dogs with IBD compared to a healthy population. A cutoff value of >0.14 for the MLR was identified with an area under the curve (AUC) of 0.839 and a 95% confidence interval (CI). The cutoff value for the PLR was found to be 131.6 (AUC = 0.675 and 95% CI) and the monocyte count cutoff was set at >370 cells/dL (100% specificity).

In our study, the monocyte count increased in group C at T60, possibly indicating worsening inflammation since this group was taking the placebo. The results for the monocyte count, PLR, and MLR were consistent with the cutoff values identified for IBD.

On the other hand, fecal concentrations of LA, SCFAs, and BCFAs are often linked to gut inflammation and dysbiosis [1,37,38]. Fecal lactate is an indicator of gut pH, and a study by Blake et al. (2019) [39] found elevated concentrations of this metabolite in dogs with chronic enteropathy. The study established a reference range for normality in healthy dogs of 1 to 7 mMol/L. In our study, dogs had fecal lactic acid concentrations within this reference. The mild state of the disease and the homogeneity of the study groups may justify this finding, in contrast to the variability observed in the referenced study. Further long-term and controlled studies might provide a better understanding.

None of the treatments interfered in the fecal concentrations of the evaluated bacterial metabolites, despite the differences found in bacterial taxa growths. Our findings are consistent to those of Swanson et al. [40] whose similarly did not find differences in fecal bacteria metabolites in dogs supplemented with prebiotics, contrary to expectations. According to their study, the lack of alteration might be attributed to the efficient absorption of SCFAs and BCFAs by colonocytes, which positively affected the growth of some bacteria without altering their concentration in feces. Thus, analysis of SCFAs and BCFAs in blood could provide different results in future studies.

Regarding the abundances of the evaluated taxa, the results exhibit the complex interpretation of dysbiosis modulation. According to the literature, the reduction in SCFA-producing bacteria, such as those from phylum Firmicutes (Erysipelotrichia, Clostridia, and Faecalibacterium) and Bacteroidetes, and the increase in the population of Proteobacteria are consistent findings in dogs with untreated IBD [40]. In a review published by Mukhopadhya et al. (2012) [38], the authors proposed that the phylum Firmicutes can be considered to be a “protective” group and the phylum Proteobacteria can be considered to be a “harming” group in human patients with IBD.

Results of this study indicate that treatments A and B promoted the growth of the phylum Firmicutes, with treatment A additionally resulting in an increase in the population of Bacteroidetes. The Firmicutes/Bacteroidetes ratio serves as a reference for assessing dysbiosis in human patients with IBD [40,41] and its reduction is considered to be undesirable.

According to You et al. (2021) [42], normal Bacteroidetes counts are around 27.73% in healthy dogs. Our results show lower percentages of these bacteria and corroborate the expected dysbiosis. Notably, treatment A led to an increase in Bacteroidetes, whereas treatment B decreased their population. Treatment C was different compared to both A and B.

When we analyze Firmicutes individually, the mean relative abundances are expected to be around 92% [43,44], which was not observed at T0 in any of the groups. However, by T60, groups A and B showed an increase to around this expected level, whereas group C exhibited a decrease, indicating a beneficial effect of the prebiotics compared to the negative evolution observed with the placebo. 

As previously mentioned, Proteobacteria are considered to be a “harming” group of bacteria in humans with IBD [41]. Specifically, the growth of Enterobacteriaceae is the hallmark of dysbiosis that is associated with many GI diseases [11]. Supplementation with 1.3, 1.6—beta-glucans has increased these populations, which is a negative outcome from this perspective. However, Proteobacteria and Enterobacteriaceae decreased in the control group (by 89%), contrary to expectations. Therefore, conclusions regarding these changes are still conflicting.

According to Omori et al. (2017) [45], dysbiosis is characterized by a decrease in SCFA-producing bacteria (Firmicutes and Bacteroidetes) and an increase in Actinobacteria and Proteobacteria. In our results, despite minor changes in Proteobacteria, the growth of Actinobacteria was controlled by both prebiotics and the placebo group exhibited detrimental changes. This suggests a positive modulation of microbiota dynamics using prebiotics that did not occur in the dogs that did not receive it, potentially indicating a marker for the negative progression of dysbiosis over time and the possibility of disease relapse. 

Ruminococcus are decreased in dogs with acute hemorrhagic diarrhea and have a discriminatory power between healthy and IBD dogs. Since they are SCFA-producing bacteria, higher counts are preferable [8,11], and an increase in this population was only observed in treatment A. 

It is important to address that, despite the individual changes mentioned, the mechanisms of quorum sensing are not being considered and they are still under study [46,47]. Therefore, these results must be interpreted with caution. 

Research by Perini et al. (2020) [48] has demonstrated that a period of 60 days is feasible and sufficient in order to observe changes in some variables of GI health in healthy dogs. The authors of this study believe that, in chronic diseases, where inflammation plays an important role in perpetuating dysbiosis, more time might be necessary to observe significant and relevant modifications.

In these situations, the Shannon index and OTU counts may provide a better insight into this analysis. These indexes are used to evaluate alpha diversity in samples and greater values indicate greater the diversity, evenness (Shannon), and richness (OTUs) of a sample. According to this definition, diversity and evenness were improved in group A, suggesting and improvement of dysbiosis since, according to Petersen and Round (2014) [49], the loss of richness and diversity is a hallmark of dysbiosis.

Jackson and Jewell (2020) [50] also found that adding fiber bundles to the food of dogs with chronic enteritis improved their alpha diversity as well; however, they also found differences in the production of SCFAs, differently from this study, even with a lower period of supplementation (4 weeks). We believe that this result may be due to the composition of the offered food (hydrolyzed meat or grain-rich food).

Beta diversity analysis indicated a different microbiome in group C despite the prebiotic intake. Minamoto et al. (2015) [1] identified differences in beta diversity between healthy and IBD dogs and, unexpectedly, this difference may be a possible limitation of our study, despite the fact that dogs were clinically homogenous. As a practical observation of our study, despite all that was previously discussed, among the 18 dogs, all of them remained clinically controlled for a minimum of six months with no alterations in their pharmacological protocols.

At the time the study was conducted, the dysbiosis index was still under development, and was not included for administrative reasons. However, following its release, this index emphasizes that not every instance of dysbiosis leads to clinical manifestations. The dysbiosis index provides a means to evaluate disease progression or remission. Therefore, we believe it could be applied in this context once we determine the optimal product dosage (or intake time), or even in longer studies involving dogs with at least moderate disease severity, to observe substantial changes and clinical outcomes. 

Understanding the effects of a prebiotic on each bacterium is also essential as fundamental research to comprehend the effects of a single nutrient or nutraceutical within the complex environment, such as the microbiota of diseased animals, before investigating these effects in multivariate scenarios. The primary question of our study was whether beta-glucans or MOSs have any impact on the gut microbiota of dogs with chronic intestinal disease at this specific dosage and during this specific timeframe. While some studies have attempted to examine broader clinical effects in these animals, the results remain uncertain. Dogs with inflammatory bowel disease can experience relapses and may require additional medications, with variations in severity and dietary intake. To isolate the effects of the prebiotic itself, we made significant efforts to gather a group with uniform disease severity. Otherwise, any lack of observed effects could be attributed to the differing disease severity among the dogs rather than an insufficient intake of beta-glucan. Having established a specific dosage and duration, future research could build upon this foundation to explore more complex scenarios.

## 5. Conclusions

The prebiotic intake was well-tolerated by all dogs and led to important shifts in the microbiota that improved microbiota diversity and evenness. Further long-term studies are required to better understand the dynamics of the microbiota in a homogenous group of dogs with moderate IBD and to evaluate clinical outcomes.

## Figures and Tables

**Table 1:** Prebiotics’ chemical composition and solubility.

Macrogard^®^ *	
Beta-glucan (g/100 g)	60.0
Fat (g/100 g)	18.0
Protein (N × 6.25) (g/100 g)	8.0
Ash	10.0
Solubility in water	7.9
Prebiotic blend	
Beta-glucan (g/100 g)	25.0
MOS (g/100 g)	20.0
MOS solubility in water	100.0

* Macrogard^®^, Biorigin, Lençóis Paulistas, Sao Paulo, Brazil.

**Table 2:** Means and standard error of the mean (SEM) of the blood variables.

	Groups					*p*-Value		
	A	B	C	SEM	Ref ^1^	Treatment	**Time**	**Treatment × Time**
Albumin (g/dL)	3.92	4.12	3.56	0.176	2.3–3.8	0.1015	0.1082	0.8482
Total protein (g/dL)	6.87	6.98	6.34	0.289	5.3–7.6	0.2744	0.4543	0.6651
ALT (U/L)	37.55	21.65	24.00	5.856	10–88	0.1513	0.1418	0.1880
Alkaline phosphatase (U/L)	250.50	68.83	48.30	85.104	20–150	0.2143	0.6185	0.5766
Urea (mg/dL)	32.60	29.40	39.27	4.835	20–40	0.3635	0.0265	0.1308
Creatinine (mg/dL)	0.92	1.01	0.83	0.072	0.7–1.4	0.2695	0.9872	0.8450
Triglycerides (mg/dL)	275.03	96.43	100.95	69.994	40–169	0.1381	0.9931	0.9888
Cholesterol (mg/dL)	278.90 ^A^	164.69 ^B^	207.09 ^AB^	26.852	125–270	0.0273	0.9801	0.9469
Cobalamin (ng/L)	700.17	511.67	428.58	79.859	252–836 ^2^	0.0781	0.7180	0.9386
Folate (nmol/L)	45.58	35.50	35.00	5.559	7–39 ^2^	0.3420	0.1976	0.4427
CRP (mg/dL)	63.61	9.37	33.93	32.406	0–10	0.5108	0.1646	0.4455
Spec (ng/mL)	166.83	98.33	55.51	37.412	<200	0.1395	0.0494	0.1266
TLI (mcg/L)	30.66	24.25	21.41	4.918	9–50	0.4167	0.8689	0.0887
Total leucocyte (/μL)	8482.50	10,203.00	9175.00	851.060	6.000–15.000	0.3679	0.4915	0.2606
Lymphocytes (/μL)	1789.50	1679.42	2169.83	359.310	1.500–5.000	0.6064	0.9219	0.3593
Monocytes (/μL)	672.33	396.17	468.37	63.758	0–800	0.0129	0.6250	0.0226
Eosinophil (/μL)	347.50	592.00	543.00	164.290	0–1.300	0.5512	0.5671	0.2877
Basophil (/μL)	69.16	39.16	47.50	27.937	0–140	0.7394	0.5324	0.5025
Platlets (10^3^/μL)	473.08 ^A^	289.67 ^B^	407.33 ^AB^	41.422	200–600	0.0014	0.4776	0.5747

ALT = alanine aminotransferase; CRP = C reactive protein; Spec = canine pancreas-specific lipase; TLI = trypsin-like immunoreactivity; A: 1.3/1.6 beta-glucan; B: MOS + 1.3/1.6 beta-glucan; C: placebo; ^1^ Kaneko (2008) [23] reference values except for folate and cobalamin; ^2^ Suchodolski; Steiner (2003) [24] reference values; Different capital letters on the same line differ by Tukey’s test (*p* < 0.05).

**Table 3:** Mean and standard error of the mean (SEM) of the alpha diversity indices, Shannon, Simpson, and OTUS at T0 and T60 in the experimental groups. A: 1.3/1.6 beta-glucan; B: 1.3/1.6 beta-glucan + MOS; C: placebo.

		Time			
	Groups	T0		T60	
		Average	SEM	Average	SEM
Shannon	A	2.34 ^Ab^	0.28	2.87 ^Aa^	0.28
	B	2.64 ^Aa^	0.28	2.29 ^Aa^	0.28
	C	2.48 ^Aa^	0.28	2.72 ^Aa^	0.28
					
OTUS	A	52.96 ^Aa^	3.87	57.61 ^Aa^	3.87
	B	54.08 ^Aa^	3.87	48.20 ^Aa^	3.87
	C	44.71 ^Aa^	3.87	61.95 ^Aa^	3.87
					
Simpson	A	3.33 ^Aa^	0.80	4.88 ^Aa^	1.05
	B	6.00 ^Aa^	2.06	2.73 ^Aa^	0.32
	C	4.11 ^Aa^	0.73	4.17 ^Aa^	0.81

Means followed by the same capital letters in a column and lower case letters on the lines do not differ by the Tukey–Kramer test (*p* ≤ 0.05).

## Data Availability

Data are contained within the article.
